# Diagnostic Value of C-Reactive Protein in Discrimination between Uncomplicated and Complicated Parapneumonic Effusion

**DOI:** 10.3390/diagnostics10100829

**Published:** 2020-10-15

**Authors:** Yana Kogan, Edmond Sabo, Majed Odeh

**Affiliations:** 1 Department of Internal Medicine A, Bnai Zion Medical Center, Haifa 31048, Israel; janakgan37@gmail.com; 2 Pulmonary Division, Carmel Medical Center, Haifa 31048, Israel; 3 Faculty of Medicine, Technion—Israel Institute of Technology, Haifa 31048, Israel; EdmondSa@clalit.org.il; 4 Institute of Pathology, Carmel Medical Center, Haifa 31048, Israel

**Keywords:** C-reactive protein, parapneumonic effusion, uncomplicated, complicated

## Abstract

Objectives: The role of serum C-reactive protein (CRPs) and pleural fluid CRP (CRPpf) in discriminating uncomplicated parapneumonic effusion (UCPPE) from complicated parapneumonic effusion (CPPE) is yet to be validated since most of the previous studies were on small cohorts and with variable results. The role of CRPs and CRPpf gradient (CRPg) and of their ratio (CRPr) in this discrimination has not been previously reported. The study aims to assess the diagnostic efficacy of CRPs, CRPpf, CRPr, and CRPg in discriminating UCPPE from CPPE in a relatively large cohort. Methods: The study population included 146 patients with PPE, 86 with UCPPE and 60 with CPPE. Levels of CRPs and CRPpf were measured, and the CRPg and CRPr were calculated. The values are presented as mean ± SD. Results: Mean levels of CRPs, CRPpf, CRPg, and CRPr of the UCPPE group were 145.3 ± 67.6 mg/L, 58.5 ± 38.5 mg/L, 86.8 ± 37.3 mg/L, and 0.39 ± 0.11, respectively, and for the CPPE group were 302.2 ± 75.6 mg/L, 112 ± 65 mg/L, 188.3 ± 62.3 mg/L, and 0.36 ± 0.19, respectively. Levels of CRPs, CRPpf, and CRPg were significantly higher in the CPPE than in the UCPPE group (*p* < 0.0001). No significant difference was found between the two groups for levels of CRPr (*p* = 0.26). The best cut-off value calculated by the receiver operating characteristic (ROC) analysis for discriminating UCPPE from CPPE was for CRPs, 211.5 mg/L with area under the curve (AUC) = 94% and *p* < 0.0001, for CRPpf, 90.5 mg/L with AUC = 76.3% and *p* < 0.0001, and for CRPg, 142 mg/L with AUC = 91% and *p* < 0.0001. Conclusions: CRPs, CRPpf, and CRPg are strong markers for discrimination between UCPPE and CPPE, while CRPr has no role in this discrimination.

## 1. Introduction

Parapneumonic effusion (PPE) is an accumulation of exudative pleural fluid that occurs in association with an ipsilateral pulmonary infection, mainly pneumonia, and may also accompany lung abscess and infected bronchiectasis. PPEs are the most common exudative pleural effusions, and are present in 20% to 54% of patients with bacterial pneumonia [1,2]. Based on the characteristics of a diagnostic thoracentesis, PPE can be classified into uncomplicated PPE (UCPPE), or complicated PPE (CPPE). UCPPEs are free flowing effusions, not infected, have a pH level greater than 7.2, a glucose level greater than 60 mg/dL, and lactate dehydrogenase (LDH) level less than 1000 IU/L. CPPE usually are infected, have a pH level less than 7.2, a glucose level less than 60 mg/dL, and LDH level greater than 1000 IU/L. These effusions are initially thin and serous, but become more purulent as the disease progresses. The natural course of a CPPE is to develop a single loculus or multiple loculations, and to progress to empyema when the effusion becomes thick and turbid, which represents the end stage of a CPPE. Additionally, large (≥1/2 hemithorax) free flowing PPE, and a PPE accompanied with thickened pleura are considered as CPPE. Most of the PPEs are UCPPEs (about 70%) and resolve spontaneously without specific therapy other than antibiotic treatment. The minority (about 30%) are CPPEs that follow a complicated course requiring, in addition to antibiotic treatment, more aggressive intervention, such as chest tube thoracostomy or pleural space decortication. Patients with CPPE have a significantly higher morbidity and mortality rate [1,2,3,4,5,6,7,8,9,10,11,12,13,14].

C-reactive protein (CRP) is an acute phase reactant synthesized and secreted in the liver by the hepatocytes. Its production is induced by systemic inflammation of either infectious or noninfectious origin, and by tissue injury. An increasing number of studies have reported that both serum CRP (CRPs) and pleural fluid CRP (CRPpf) can play a role in diagnosing PPE and differentiating UCPPE from CPPE, although with moderate diagnostic ability [11,15,16,17,18,19,20,21,22,23,24]. Most of these studies were conducted on small cohorts of patients, and the efficacy rate of these two parameters differs among these studies [11,15,16,17,18,19,20,21,22,23,24]. Until now, the diagnostic value of CRPs and the CRPpf gradient (CRPg), and CRPpf to CRPs ratio (CRPr) in the discrimination between UCPPE and CPPE has not been assessed.

The aim of this study, which was conducted on a cohort of patients with PPE (146 patients)—larger than the cohorts of most other previous studies in this regard—who were admitted to our Department of Internal Medicine, at Bnai Zion Medical Center between January 2000 and October 2016, is to assess the diagnostic value of CRPs, CRPpf, CRPg and CRPr in the discrimination between UCPPE and CPPE.

## 2. Materials and Methods

### 2.1. Patients

The study population consisted of 146 patients with PPE; 86 patients aged 24–91 years UCPPE, and 60 patients aged 30–91 years were with CPPE. Effusion was considered UCPPE when there was acute febrile illness with purulent sputum, pulmonary infiltrate, and responsiveness to antibiotic treatment, in the absence of other diseases causing pleural effusion, and with no direct or indirect evidence of bacterial invasion of the effusion. Effusion was considered CPPE in the presence of pneumonia confirmed clinically and radiographically in the absence of other diseases causing pleural effusion, with one or more of the following indicators of bacterial invasion of the effusion: bacteria in the Gram stain or culture of the pleural fluid, presence of loculations in the pleural cavity, thickening of the parietal pleura, pH of the pleural fluid <7.2, the PPE is empyema, or the effusion is large (≥1/2 hemithorax).

### 2.2. Methods

Data collection was completed from the patients’ charts. Only patients with definitive diagnosis of their PPE as being UCPPE or CPPE, with measurement of CRPs and CRPpf were included in the study. Patients suffering from other diseases that can affect the serum level of C-reactive protein were not included in the study. PPE was considered UCPPE or CPPE according to the above-mentioned criteria. CRPs and CRPpf levels were measured on a Cobas c 501 analyzer of Roche Diagnostics by C-Reactive Protein Gen.3 assay. The method is a particle enhanced immunoturbidometric where human CRP agglutinates with latex particles coated with monoclonal anti-CRP antibodies. The aggregates are determined turbidometrically at 546 nm, where the measuring range of the assay is between 0.3–350 mg/L, and the normal range is ≤5 mg/L. The study was conducted in accordance with the Declaration of Helsinki, and the protocol was approved by the Ethics Committee of Bnai Zion Medical Center (0107-16-BNZ, 25 October 2016. CRPg was calculated as CRPs—CRPpf, and CRPr was calculated as CRPpf/CRPs.

### 2.3. Statistical Analysis

Descriptive statistical values are presented as means ± standard deviation (SD) of means, and 95% confidence intervals (CIs). The Kolmogorov–Smirnov test was done to evaluate the normality of the data. Comparisons between parametric groups were completed using the unpaired Student’s *t*-test. The *p*-values were corrected for multiple comparisons using Bonferoni correction. Receiver operating characteristics (ROC) analysis was used to detect the best cut-off values (i.e., those with the highest total accuracy) for separating UCPPEs from CPPEs. Sensitivity, specificity, positive predictive value (PPV), negative predictive value (NPV), total accuracy, odds ratio, and area under the ROC curve (AUC) were calculated. The significance of the best cut-off values was evaluated using the χ^2^ test or the Fisher’s exact test as needed. Two tailed *p*-values of ≤ 0.5 were considered statistically significant.

## 3. Results

The UCPPE group included 86 patients, and the CPPE group included 60 patients. In all patients the CRPs level was higher than that of CRPpf. The mean age of the CPPE group was significantly higher than in the UCPPE group: 74.1 ± 13.6 years vs. 65.9 ± 18.1 years, respectively (*p* < 0.003) (Table 1).

**Figure 1:**
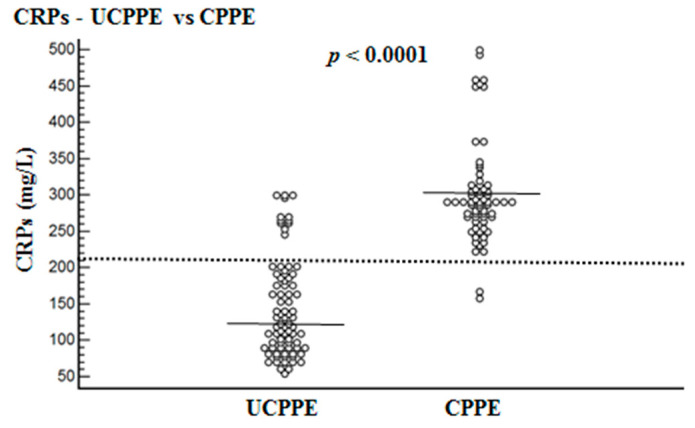
Serum CRP levels (CRPs), and their means for uncomplicated parapneumonic effusion (UCPPE) group (145.3 mg/L) and complicated parapneumonic effusion (CPPE) group (302.2 mg/L, and best cut-off value (211.5 mg/L) for discrimination between UCPPE and CPPE. For both parameters *p* < 0.0001.

**Figure 2:**
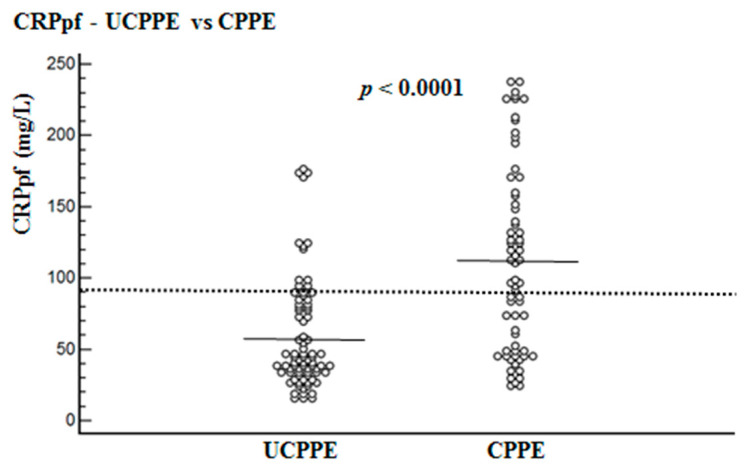
Pleural fluid CRP levels (CRPpf) and their means for uncomplicated parapneumonic effusion (UCPPE) group (58.5 mg/L) and complicated parapneumonic effusion (CPPE) group (112.0 mg/L), and best cut-off value (90.5 mg/L) for discrimination between UCPPE and CPPE. For both parameters *p* < 0.0001.

**Figure 3:**
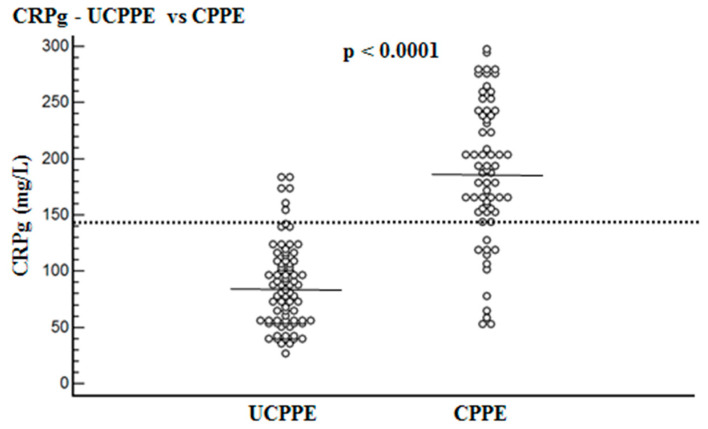
Serum and pleural fluid CRP gradient (CRPg) levels for uncomplicated parapneumonic effusion (UCPPE) group (86.9 mg/L) and complicated parapneumonic effusion (CPPE) group (188.3 mg/L), and best cut-off value (142 mg/L) for discrimination between UCPPE and CPPE. For both parameters *p* < 0.0001.

Mean levels of CRPs, CRPpf, CRPg and CRPr are presented in Table 1 and Figure 1, Figure 2 and Figure 3. The mean level of CRPs was significantly higher in the CPPE group than in the UCPPE group: 302 ± 75.6 mg/L (95% CI: 283.9–320.4) vs. 145.3 ± 67.6 mg/L (95% CI: 130.5–160.1), respectively (*p* < 0.0001) (Table 1; Figure 1). The mean level of CRPpf was significantly higher in the CPPE group than in the UCPPE group: 112 ± 65 mg/L (95% CI: 96–128) vs. 58.5 ± 38.5 mg/L (95% CI: 19.2–85.1), respectively (*p* < 0.0001) (Table 1; Figure 2). The mean level of CRPg was significantly higher in the CPPE group than in the UCPPE group: 188.3 ± 62.3 mg/L (95% CI: 173–203.6) vs. 86.9 ± 37.3 mg/L (95% CI: 48.6–99.2), respectively (*p* < 0.0001) (Table 1; Figure 3). Mean level of CRPr in the CPPE group and in the UCPPE group was 0.36 ± 0.19 mg/L (95% CI: 0.25–0.39) and 0.39 ± 0.11 (95% CI: 0.31–0.47), respectively. No significant difference was found between the two groups (*p* = 0.26) (Table 1).

**Figure 4:**
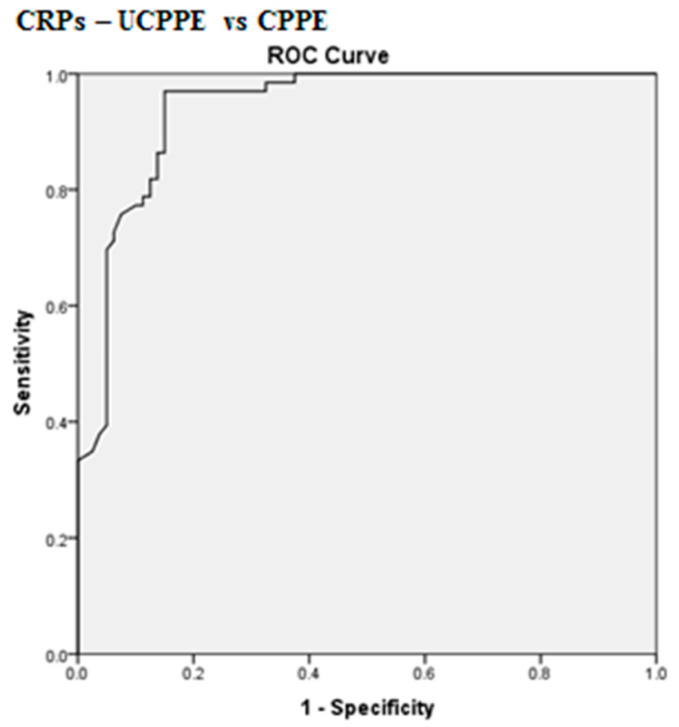
Receiver operating characteristics (ROC) curve of best cut-off value of CRPs for discrimination between UCPPE and CPPE. CRPs best cut-off value = 211.5 mg/L, area under the ROC curve (AUC) = 94% (95% CI: 90–97.7), sensitivity = 97%, specificity = 85.0%, total accuracy = 90.4%, positive predictive value (PPV) = 84.2%, negative predictive value (NPV) = 97.1%, odds ratio = 181.3 (risk for CPPE when CRPs value > 211.5 mg/L), *p* < 0.0001.

**Figure 5:**
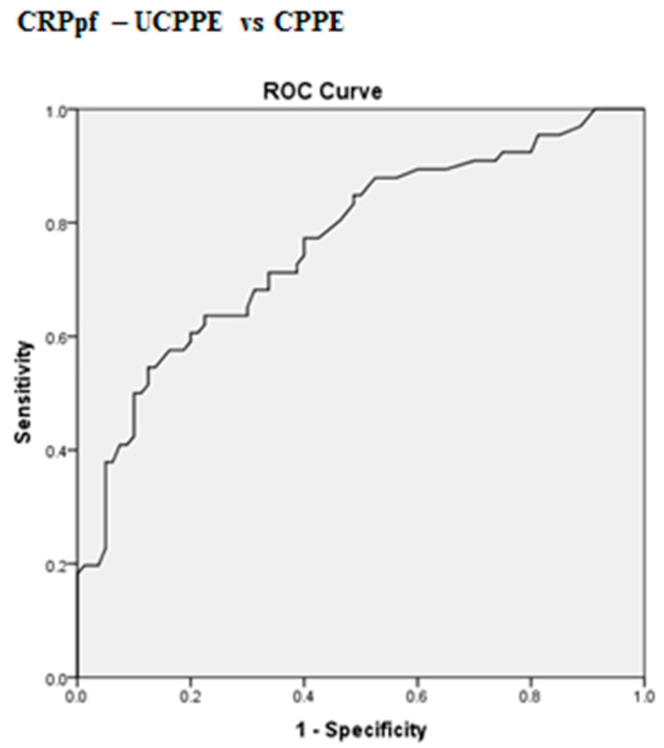
ROC curve of best cut-off value of CRPpf for discrimination between UCPPE and CPPE. CRPpf best cut-off value = 90.5 mg/L, AUC = 76.3% (95% CI: 68.5–84.1), sensitivity = 57.6%, specificity = 83.8%, total accuracy = 72%, PPV = 74.5%, NPV = 70.5%, odds ratio = 6.9 (risk for CPPE when CRPpf value > 90.5 mg/L), *p* < 0.0001.

**Figure 6:**
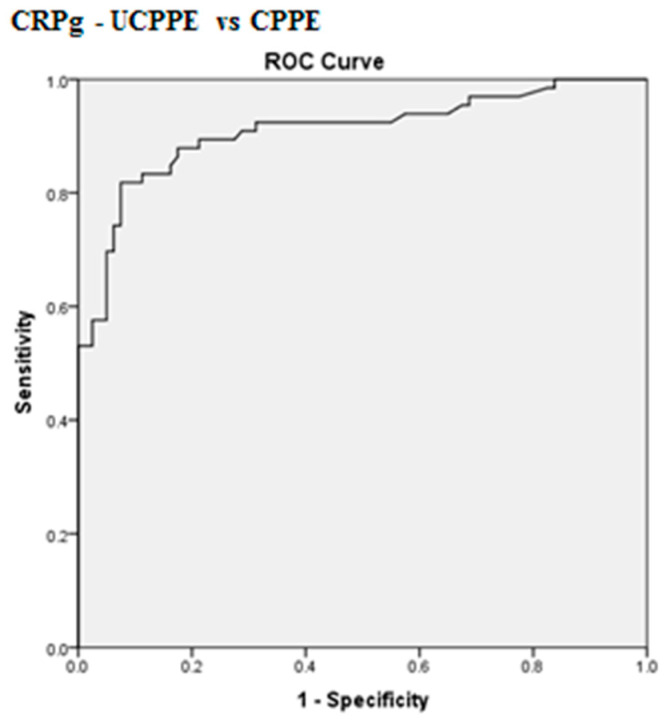
ROC curve of best cut-off value of CRPg for discrimination between UCPPE and CPPE. CRPg best cut-off value = 142 mg/L, AUC = 91% (95% CI: 85.8–96.0), sensitivity = 90%, specificity = 86%, total accuracy = 88%, PPV = 82%, NPV = 92.5%, odds ratio = 55.5 (risk for CPPE when CRPg value > 142 mg/L), *p* < 0.0001.

The best cut-off values for CRPs, CRPpf and CRPg, which were calculated by the ROC analysis, for discrimination between UCPPE and CPPE together with their relevant statistical parameters are presented in Figure 1, Figure 2, Figure 3, Figure 4, Figure 5 and Figure 6.

The best cut-off value for CRPs was 211.5 mg/L, with a sensitivity of 97%, specificity of 85%, total accuracy of 90.4%, AUC of 94%, and *p* < 0.0001 (Figure 1 and Figure 4). The best cut-off value for CRPpf was 90.5 mg/L, with a sensitivity of 76.3%, specificity of 83.8%, total accuracy of 72%, AUC of 76.3%, and *p* < 0.0001 (Figure 2 and Figure 5).

The best cut-off value for CRPg was 142 mg/L, with a sensitivity of 90%, specificity of 86%, total accuracy of 88%, AUC of 91%, and *p* < 0.0001 (Figure 3 and Figure 6).

## 4. Discussion

The main decision to make in managing a patient with PPE is whether to insert a chest tube. Because a delay in instituting proper pleural drainage in such patients substantially increases morbidity and mortality, it is important to determine—as early as possible—whether a PPE is complicated. If the PPE is empyema: loculations will be present in the pleural cavity, the Gram stain or culture of the pleural fluid will be positive, the effusion will be large (≥1/2 hemithorax), and thickening of the parietal pleura will be present, tube thoracostomy is absolutely indicated regardless of the pH value, glucose and LDH levels [1,2,3,4,5,6]. However, the diagnostic sensitivity of the Gram stain or culture of the pleural fluid for CPPE is low, with only about 40% of these effusions being positive [3,7,8]. In cases where the effusion is not empyema, not loculated, not large, with the Gram stain and culture of the pleural fluid being negative, and where the parietal pleura is not thickened, the decision to insert a chest tube depends mainly on the pH value which was found to be superior to values of glucose and LDH in predicting CPPE [8,9,10]. Furthermore, some studies have shown that a pleural fluid level of LDH > 1000 IU/L and a glucose level < 60 mg/dL do not improve the diagnostic yield of CPPE and will only be used if the pH cannot be determined [9,10]. Although according to the existing guidelines, a pleural fluid pH < 7.2 is probably the most accepted biochemical characteristic for predicting CPPE, it lacks sufficient sensitivity (i.e., effusions with value of pleural fluid pH > 7.2 may not resolve without a chest tube insertion). Furthermore, measurement of pH is influenced by the sample collection method, and may vary significantly between different locules in multiloculated and infected PPEs [11,12,13,14].

The role of CRPs and CRPpf in the discrimination between UCPPE and CPPE has comprised a hot research topic in recent years. An increasing number of studies have reported that both CRPs and CRPpf can play a role in discriminating UCPPE from CPPE, although with moderate diagnostic ability [11,15,16,17,18,19,20,21,22,23,24]. Most of these studies were conducted on small cohorts of patients, and the efficacy rate of these two parameters differs among these studies [11,15,16,17,18,19,20,21,22,23,24].

The results of our study, which was conducted on a cohort of 146 patients with PPE, larger than cohorts of most other previous studies, demonstrate that both CRPs and CRPpf are useful markers for discrimination between UCPPE and CPPE (Table 1; Figure 1 and Figure 2), and their efficacy rate in this discrimination is much more higher than that of other previous studies [11,15,16,17,18,19,20,21,22,23,24]. With a best cut-off value of 211.5 mg/L for CRPs, the discrimination between the two groups was excellent (Figure 1 and Figure 4). With a best cut-off value of 90.5 mg/L for CRPpf, the discrimination between the two groups was good (Figure 2 and Figure 5). These results are in agreement with results of most previous studies in this regard [11,15,16,17,18,19,20,21,22,23,24], but are stronger and more accurate in indicating a strong role of CRPpf and particularly CRPs in the discrimination between UCPPE and CPPE.

This is the first time that the role of CRPg and CRPr in discrimination between UCPPE and CPPE has been investigated. The rationales for testing the value of CRPg and CRPr for discrimination between UCPPE and CPPE are that both markers were not tested for this discrimination before. Furthermore, CRPr was tested for the discrimination between transudative and exudative PE, and between various types of exudative plural effusions in some previous studies, but with variable results [25,26,27,28]. The gradient of another marker (tumor necrosis factor-α) was tested for the discrimination between UCPPE and CPPE in one of our previous studies, and was found to have very good diagnostic efficacy [29]. The results of our study demonstrate that CRPg is a very useful marker for the discrimination between UCPPE and CPPE (Table 1; Figure 3). With a best cut-off value of 142 mg/L for CRPg, the discrimination between the two groups was excellent (Figure 3 and Figure 6). These results indicate, for the first time, a strong role of CRPg in the discrimination between UCPPE and CPPE, even better than that of CRPpf. No significant difference between the two groups was found for CRPr (Table 1) indicating that there is no role of CRPr in the discrimination between UCPPE and CPPE. The reason why CRPg, but not CRPr, could play a role in this discrimination is a mathematical issue. CRP is produced solely in the liver, and its level in the pleural fluid depends on its level in the blood, and increases in the same manner of its increase in the blood. The CRPr does not change significantly when the CRPpf increases with the increase in the CRPs, while the CRPg level increases significantly with a parallel increase in CRPs and CRPpf.

The mean age of the CPPE group was significantly higher than that of the UCPPE group (74.1 ± 13.6 years vs. 65.9 ± 18.1 years, respectively, *p* < 0.003). Although CRPs may increase with age, this increase is very small and remains within the normal range of CRPs which is below 5 mg/L [30]. For this reason, age does not significantly influence the mean levels of CRPs in the UCPPE and CPPE groups which were 145.3 ± 67.6 mg/L and 302.2 ± 75.6 mg/L, respectively. Thus, the results of CRPs obtained in the study are not biased by age.

This study has one limitation; it is a retrospective study.

## 5. Conclusions

The results of our study, which was conducted on cohort of patients larger than that in most previous studies, demonstrated a strong role of CRPs and CRPpf in the discrimination between UCPPE and CPPE. It also demonstrated, for the first time, a strong role of CRPg and no role of CRPr in this discrimination. These results encourage further prospective studies conducted on large cohorts of patients in order to further investigate the validity of the role of CRPs, CRPpf, and CRPg in the discrimination between UCPPE and CPPE.

## Figures and Tables

**Table 1:** Mean ± SD of age, and mean ± SD and 95% CI of means of levels of CRPs, CRPpf, CRPg, and CRPr of UCPPE group and CPPE group.

Parameter	UCPPE (*n* = 86)	CPPE (*n* = 60)	*p*
Age (years)	65.9 ± 18.1	74.1 ± 13.6	<0.003
CRPs (mg/L) 95% CI	145.3 ± 67.6 130.5–160.1	302.2 ± 75.6 283.9–320.4	<0.0001
CRPpf (mg/L) 95% CI	58.5 ± 38.5 19.2–85.1	112.0 ± 65.0 96.0–128.0	<0.0001
CRPg (mg/L) 95% CI	86.9 ± 37.3 48.6–99.2	188.3 ± 62.3 173.0–203.6	<0.0001
CRPr 95% CI	0.39 ± 0.11 0.31–0.47	0.36 ± 0.19 0.25–0.39	0.26

SD: Standard deviation, CI: Confidence interval, CRPs: Serum C-reactive protein, CRPpf: Pleural fluid CRP, CRPg: CRPs and CRPpf gradient, CRPr: CRPpf to CRPs ratio, UCPPE: Uncomplicated parapneumonic effusion, CPPE: Complicated parapneumonic effusion.
